# Hypertrophia of Papilla of the Tongue

**Published:** 1861-11

**Authors:** 


### HYPERTROPHIA OF THE PAPILLA OF THE TONGUE.

In February last we had occasion to visit a patient—a child about four years old,—with an affection of the tongue, which we designated as above. It possessed apparently a good constitution, and never had any sickness, except an abscess, which was formed just back of the right ear, which involved the neighboring parts to a considerable extent. This was when the child was about three years old, and it continued a few months, when the child was entirely restored. The tongue was coated over from the tip to its base, and completely from side to side, with what seemed to be enlarged or elongated papilla, to the depth of about one line. The termination at the border was abrupt, the thickening about as great there as in the centre, except that part at the base of the tongue, where it was quite thin. At the sides and the point of the tongue, the growth folded over somewhat.

On June 25th I saw the case again, the growth was evidently being removed, the base of the tongue was cleared about two lines in extent, the sides and tip also showed signs of clearing; the growth is very white ; the tongue where cleared has a beautiful healthy appearance. The affection occasions no difficulty to the child, neither general or local, so far as health is concerned; its appetite is normal, the acts of mastication and deglutition are well performed. It does not yet talk, however ; whether this is occasioned by the affection, or is a natural tardiness we are not able to determine. The child is now, Nov. 1st, five years old. As to the cause of this affection we are entirely ignorant—do not attempt to make a supposition or a suggestion.

There is no cachectic diathesis apparent either in the child or parents. The case was for a time in the care of Dr. Greenleaf, of this city; he first saw it in February, and had charge of it two months. At first it bled freely upon being touched, and had some indication of a malignant affection, and was supposed to be so by two or three physicians who saw it. Within a few days after Dr. G. saw it, the mucus membrane of the cheeks and roof of the mouth assumed the same whitish appearance, though without the thickening exhibited upon the tongue.

The tendency to hemorrhage from the growth on the tongue was arrested by the use of persulphate of iron; after using this for a few days, changed to the perchloride of iron. Iodide of ammonia was administered three times a day for two or three weeks, with no apparent improvement of the tongue; but the mucous membrane of the cheeks and the roof of the mouth was restored to a healthy condition. Small doses of calomel were daily administered for a time without producing any apparent change upon the affected parts. Other treatment was employed but without any special result. A gradual improvement seems to be going on; the posterior third of the tongue is almost entirely clear, the margins are more free than heretofore, and the entire coatings thinner, and its surface broken up by sinuosities. The case is entirely new to us, and we should be glad to have suggestions in regard to it. T.

